# Isolation of novel sequences targeting highly variable viral protein hemagglutinin^☆^

**DOI:** 10.1016/j.mex.2015.02.005

**Published:** 2015-02-16

**Authors:** Zhiwu Xu, Jieyu Wu, Fan Feng, Xiaoxiao Zhang, Xiaoqian Ma, Man Tang, Yan Huang, Ying Zhang, Yongchang Cao, Weiguo Cao, Ran He, Ye Gao, Qiuyun Liu

**Affiliations:** a MOE Key Laboratory of Aquatic Product Safety, State Key Laboratory of Biocontrol, Biotechnology Research Center, The School of Life Sciences, Sun Yat-sen University, Guangzhou 510275, China; b School of Life Sciences, Tsinghua University, Beijing 100084, China; c Guangzhou Center for Disease Control and Prevention, Guangzhou 510440, China; d Department of Genetics and Biochemistry, Clemson University, Clemson, SC 29634, USA; e Division of Biological Sciences, University of California, San Diego, La Jolla, CA 92093, USA

**Keywords:** Hemagglutinin gene, H5N1, Novel sequences, Viral variations, Mutations, Full length gene

## Abstract

Rapid evolution is a hallmark of the viral kingdom and a major concern for developing universal vaccines. The isolation of substantial numbers of viral sequence variants at highly variable viral protein domains remains a major challenge. We previously developed a combinatorial method for the isolation of novel sequences to cope with rapid viral variations at the G-H loop of Foot and Mouth Disease virus VP1 protein [1]. Here we present a modification of that method in its application in the randomization of the hemagglutinin gene from a H5N2 virus, namely:

• removal of potentially stressful region which harbored a stretch of basic amino acids to increase the success rates of gene cloning, and to streamline the process of future engineering of novel viral variants.
• clustered randomization in a full-length gene, as the positive rate for partial gene fragment libraries was extremely low before enrichment in the previous FMDV studies.
• the use of fusion partner was avoided, which was used previously for protein expression, stabilization of clones and reduction of stresses on host cells.
• the use of Poisson distribution is proposed to approximate sequencing output to achieve cost effectiveness.

## Graphical abstract

**Figure:**
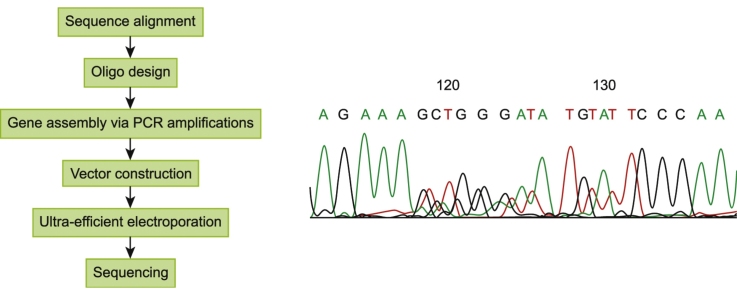

## Methods

**Fig. 1:**
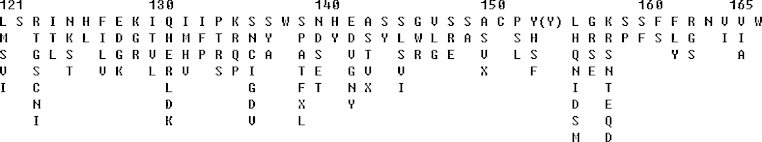
Polymorphisms at 121–165 aa regions of the H5N1 hemagglutinin proteins.

Three semi-random oligonucleotides (oligos) were designed based on sequence alignment (Table 1 and Fig. 1). DNA fragments were assembled via PCR amplification with high fidelity PrimeSTAR DNA polymerase. Meanwhile, amino acids at the cleavage site of hemagglutinin found in highly virulent H5N1 and H5N2 strains were replaced with a low pathogenic sequence. Full-length hemagglutinin gene was produced via overlapping PCR, and was inserted into the BamHI and Hind III restriction site of pBluescript SK(−) vector. A simultaneous treatment of the vector with EcoR I removed most insert-free vector DNA by a complete digestion of the vector DNA to a poorly transformed linear form [1], [2], [3]. The clones carrying HA sequences were obtained by using an ultra-efficient electroporation method. Putative positive clones were sequenced after PCR characterizations. Eventually DNA vaccine will be prepared.

## Method detail

### Reagents

DNA fragments were recovered from agarose gel slices using QIAquick gel extraction kits (QIAGEN, GmbH, Hilden, Germany) according to manufacturer’s instructional manual. All restriction enzymes and molecular standards were obtained from Takara (Dalian, China). 2-Pyrrolidone was purchased from Tokyo Kasei Kogyo Co., Ltd. (Chuo-ku, Tokyo, Japan). Ampicillin was added at a concentration of 100 μg/ml.

### Oligo design

Sequence alignment of the hemagglutinin proteins from H5N1 strains, between January, 1913 and March, 2007, was made after being retrieved from GenBank database. The consensus sequence is embedded in the semi-random design (Table 1 and Fig. 1).

### Viral variation targeted sequence isolation via random DNA technology

**Fig. 2:**
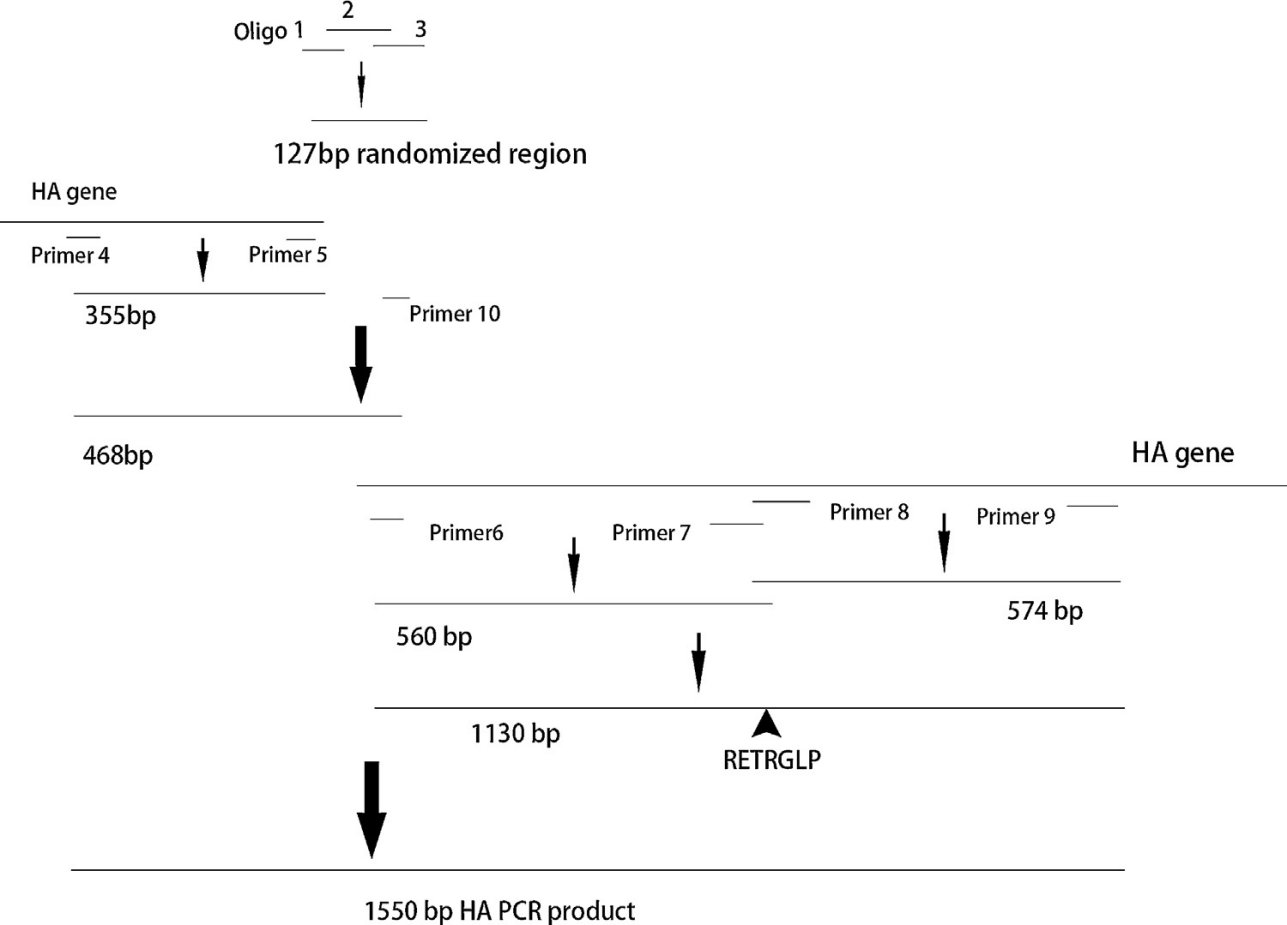
HA gene assembly via PCR amplifications. DNA fragments of different sizes were generated in a stepwise fashion, and assembled via overlapping PCR.

The full-length gene was assembled in a stepwise fashion (Fig. 2). Two microliters of oligo 2 and oligo 3 were assembled in a 20-μl reaction using PrimeSTAR DNA polymerase in the presence of 2.5% 2-pyrrolidone [4]. The PCR mixture was heated at 94 °C for 2 min, followed by 4 cycles of amplification at 94 °C for 30 s, 33 °C for 60 s and 72 °C for 20 s, and a final extension at 72 °C for 3 min, which was then placed at 4 °C for 1 h. Three microliters of oligo 1 and 2 μl of oligo 3 as well as 0.5 μl of PrimeSTAR DNA polymerase were then added. Subsequently the PCR mixture was heated at 94 °C for 2 min, followed by 15 cycles of incubation at 94 °C for 30 s, 33 °C for 60 s and 72 °C for 20 s, and a final extension at 72 °C for 3 min, which gave rise to a 127 bp subassembly product.

To generate the leftmost 355-bp subassembly product, the PCR reaction was performed with the HA gene as template, using primer 4, primer 5 and PrimeSTAR DNA polymerase in the presence of 2.5% 2-pyrrolidone (Fig. 2). The PCR mixture was heated at 94 °C for 2 min, followed by 40 cycles of amplification at 94 °C for 30 s, 30 °C for 60 s and 72 °C for 80 s, and a final extension at 72 °C for 3 min.

To synthesize the 468 bp subassembly product, the overlapping PCR reaction was conducted with the two products above as templates (Fig. 2), using primer 4 and primer 10, and PrimeSTAR DNA polymerase in the presence of 2.5% 2-pyrrolidone. The PCR mixture was heated at 94 °C for 2 min, followed by 40 cycles of amplification at 94 °C for 30 s, 30 °C for 60 s and 72 °C for 30 s, and a final extension at 72 °C for 5 min.

The PCR reaction was carried out with the HA gene as template, using either primer 6/7 or primer 8/9 and PrimeSTAR DNA polymerase in the presence of 2.5% 2-pyrrolidone. The PCR mixture was heated at 94 °C for 2 min, followed by 40 cycles of amplification at 94 °C for 30 s, 30 °C for 60 s and 72 °C for 80 s, and a final extension at 72 °C for 3 min, which gave rise to a 560-bp and a 574-bp subassembly product, respectively (Fig. 2).

The overlapping PCR reaction was performed with the 560 bp and 574 bp products, using primers 6 and 9, and PrimeSTAR DNA polymerase in the presence of 2.5% 2-pyrrolidone (Fig. 2). PCR mixture was heated at 94 °C for 2 min, followed by 35 cycles of amplification at 94 °C for 20 s, 26 °C for 60 s and 72 °C for 30 s, and a final extension at 72 °C for 5 min, which gave rise to a 1130-bp subassembly product that removed the basic amino acids in the cleavage site of HA with the insertion of RETRGLP.

To generate the final 1550-bp full-length HA PCR product, the overlapping PCR reaction was conducted with the 468 bp and 1130 bp products (Fig. 2), using primers 4, 9 and 10, and PrimeSTAR DNA polymerase in the presence of 2.5% 2-pyrrolidone. The PCR mixture was heated at 94 °C for 2 min, followed by 40 cycles of amplification at 94 °C for 30 s, 30 °C for 60 s and 72 °C for 2 min, and a final extension at 72 °C for 5 min.

0.98 μg SK(−) vector was triple digested with 12 U each of BamH I, EcoRI and Hind III for 4 h at 37 °C, followed by heat inactivation at 75 °C for 15 min. The mixture was then precipitated with ethanol and dissolved in 20 μl of double distilled water. The HA assembly products were treated likewise, and resuspended in 40 μl of double distilled H_2_O.

**Fig. 3:**
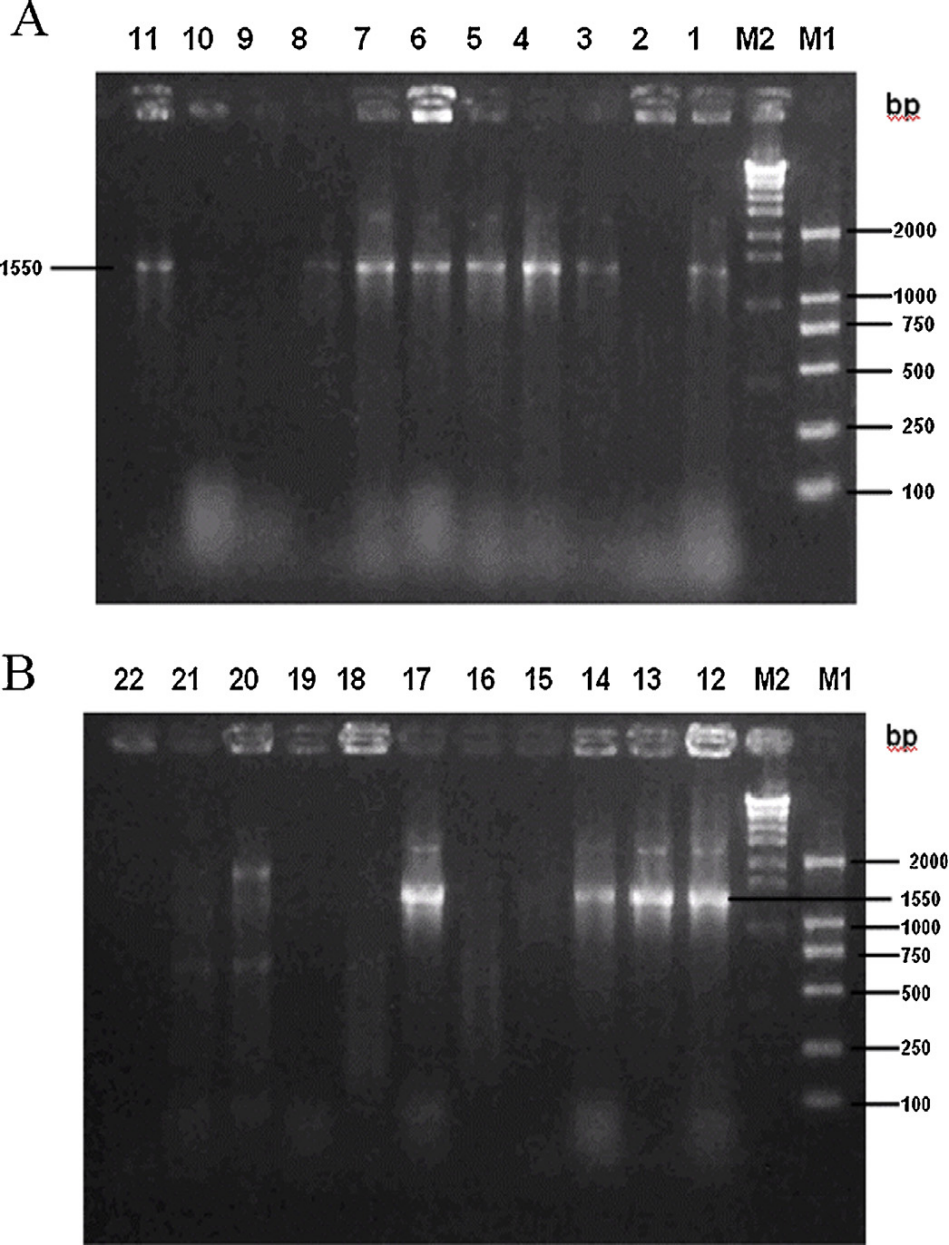
Colony PCR yielded 1550 bp products from *E. coli* transformants. M: molecular standard. Lanes 1–22 were PCR amplifications of 22 colonies.

**Fig. 4:**
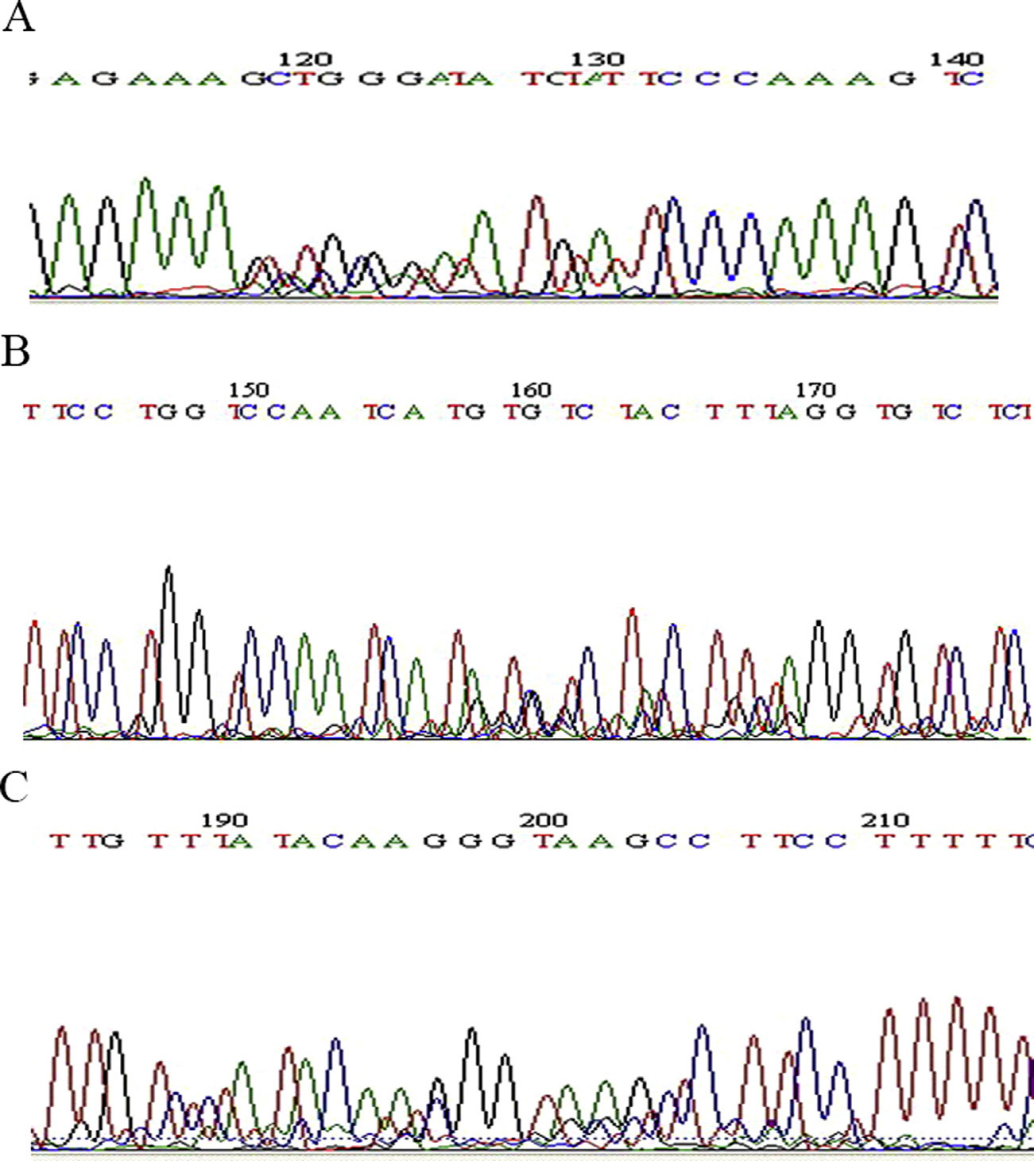
The sequencing chromatograms of the first (A), second (B) and third (C) variable regions of the transformant library pool.

The digested SK(−) vector and the HA assembly products were ligated with at an approximate molar ratio of 1:7 in the refrigerator (5 °C). The ligation mixtures were precipitated with ethanol and dissolved in 10 μl of sterile double distilled H_2_O or less, and electroporated to *E. coli* ElectroMax-DH10B strain (Invitrogen, Carlsbad, CA) [5]. Cells were plated onto LB/ampicillin plates. PCR was performed with primers 4 and 9 after microwave treatment of toothpick touched colonies [6], and 1550 bp PCR amplicons were visualized on agarose gel (Fig. 3). The positive rate was 54.5%, which was about 26-fold higher than that of the previous FMDV gene fragment libraries before the enrichment procedure [1]. The clones of *E. coli* DNA library carrying hemagglutinin gene fragment failed to grow, most likely resulted from stressful peptides expressed from the partial gene. Therefore the engineering of a full-length gene was advantageous, especially for genes from highly virulent viruses. Two of the putative positive clones were successfully sequenced and found to be novel. Bacteria were washed from the plates, and pooled, and treated as aforementioned. PCR amplicons were subsequently sequenced and shown to harbor multiple sequences (Fig. 4). The presence of virtually all the designed bases in the degenerated sites suggests that the library carried many clones.

## The use of Poisson distribution to approximate sequencing output

Due to the fact that the gene assembly process involved multiple PCR steps and gene cloning is subjected to bias such as preferential amplification of some sequences or bias during oligo synthesis [7], some sequences could be overrepresented in the sequencing stage. The use of Poisson distribution to approximate sequencing output is proposed to achieve cost effectiveness. Assuming that there are one DNA sequence redundant pair and 23 singletons in 25 positive clones initially sequenced,

$$P(X=1)=\frac{23}{N}=\lambda {\text{e}}^{-\lambda }$$

$$P(X=2)=\frac{1}{N}=\left(\frac{\lambda^{2}}{2}\right){\text{e}}^{-\lambda }$$

Then, average fold of coverage *λ* = 2/23, and total number of different positive clones *N* ≈ 289

For a further sequencing of 86 positive clones:

*P*(*X* > 0) = 1 − *P*(*X* = 0) = 1 − e^−*λ*^ = 1 − e^−(25 + 86)/*N*^ ≈ 0.32

Number of new clones could be identified in the second phase sequencing = *N* × *P*(*X* > 0) − 24 = 289 × 0.32 − 24 ≈ 68.

This is consistent with our previous calculations using our earlier formulas and result of the previous sequencing output [1] (Table 2, Table 3).

## Figures and Tables

**Table 1:** Oligos and primers used in this study.^a^

Oligos	DNA sequences
Oligo 1	GCTCTAGAAATAAACCACTTTGAGAAADHSSDWATKW TCCCAAAGTCTTCCTGGTCC
Oligo 2	CCAAAGTCTTCCTGGTCCARTCATRDSKCTWCTKYAG GTGTCTCTTCTGCTTGTY
Oligo 3	CCGAATTCACATTTCTGAAAAAGGAAGRSBYACCSDD GTATRRACAAGCAGAAGACA
Primer 4	ATGGATCCAAAAATGGATCAGATTTGCATTGGTTTCC
Primer 5	TCTCAAAGTGGTTTATTCTGCTCAATAGGTG
Primer 6	CTCCTTTTTCAGAAATGTGGTATGGCTC
Primer 7	AAATAGTCCTCTGGTTTCTCTTTGAGGGGTATTTCTGAGTCCAGT
Primer 8	AAAGAGAAACCAGAGGACTATTTGGAGCTATAGCAGGGTTTATAGAG
Primer 9	CCCAAGCTTTCATATTTGGTAAGTTCCCATTGATTCCAATTTTACTCCAC
Primer 10	GATGAGCCATACCACATTTCTGAAAAAGGAG

a Representations for degenerate bases: M = A/C, R = A/G, W = A/T, S = G/C, Y = C/T, K = G/T, V = A/G/C, H = A/C/T, D = A/G/T, B = G/C/T, N = A/G/C/T.

**Table 2:** Comparisons between our method and other methods for discovering novel viral gene variants.

Our method	Our previous method, or in vivo SElex approach, or other directed evolution methods
Working with the full length gene has eliminated the obligatory enrichment step and the use of antioxidant. Consequently the positive rate was about 26 fold higher than the previous FMDV gene fragment libraries before the enrichment procedure. Sequences obtained were ready for making DNA vaccines for animals.	Chimerization of the gene fragments was performed to allow gene expression and reduce stresses on host cells in our previous method. A post-electroporation enrichment procedure was required to increase the discovery rate of positive clones. Immunogenicity may be a problem since the peptides were only 63 residues in length.
The adoption of Poisson distribution is proposed to approximate sequencing output to achieve cost effectiveness	Our previous method did not use statistical method for projection of sequencing output.
Our method targeted protein coding gene. Protein coding regions could be under higher selection pressure than non-coding regions.	The in vivo SElex approach, or other directed evolution methods mostly targeted untranslated viral regions [8], [9], [10], [11], which are not ideal as antigenic epitopes.
Our approach can target both highly variable domains and full length proteins. It can generate both clustered and dispersed mutations. Mutations can be radical or conservative.	Error-prone PCR, DNA shuffling and pertinent approaches generated mostly dispersed point mutations [12], [13].
Hemagglutinin gene in this study was from a highly virulent H5N2 virus, which shared very high homology with its counterparts in H5N1 viruses.	To our knowledge, directed evolution has only been performed on H5N1 receptor specificity with limited number of mutations introduced [14].
The removal of a stretch of basic amino acids may be advantageous, as cationic amino acids frequently feature in antimicrobial peptides, which are also detrimental to animal cells. Vaccine development process can be streamlined with such a prior consideration	To our knowledge, no such concern has been addressed in directed evolution experiments related to Avian influenza.

**Table 3:** Troubleshooting.

Problems encountered	Solution
Fully random design did not work	Try semi-random design which is based on sequence alignment
Unable to form clones	Stressful peptides at protein terminals need to be avoided to confer less strain to the host cells. It is best that residues generally enriched in terminals of natural proteins are present in the terminals of randomized proteins and peptides.
Too many background clones harboring insert free vector	Try triple restriction digests as reported [1], [2], [3]
Plasmid yield could be very low	Use PCR to amplify genes for long term storage
Sequences may not have adapted to host perfectly, and expression in *E. coli* or yeast is low	Make protein fusions to reduce stresses, and use some other viral proteins as adjuvant when preparing protein vaccines
No colonies after electroporations	Do a calibration of electroporation efficiency; try commercially available competent cells or use highly efficient protocols [5]; under certain circumstances, withdrawal of sucrose in the protocol of the above reference yielded higher efficiencies. Our method requires ultra-high electroporation efficiency in the orders of 10^9^ to 10^10^ per microgram.
α complementation for easy visualization or protein induction during cloning	Not recommended for high expression may increase stresses to host cells
Difficulty in plasmid sequencing	Try sequencing PCR amplicons; grow clones at lower temperature or in the presence of 1–4% glucose to repress gene expression prior to PCR.
Reducing workload	Next generation sequencing can be performed, followed by primer design, PCR of the library pools and cloning.
